# TEST-RETEST RELIABILITY OF THE NIH TOOLBOX - COGNITION BATTERY OVER SHORT RETEST INTERVALS

**DOI:** 10.1093/geroni/igae098.3733

**Published:** 2024-12-31

**Authors:** Aaron Bowes, Bruno Giordani, Voyko Kavcic

**Affiliations:** Wayne State University, Detroit, Michigan, United States; University of Michigan, Ann Arbor, Michigan, United States; Wayne State University, Detroit, Michigan, United States

## Abstract

The NIH Toolbox-Cognition Battery (NIHTB-CB) is a commonly used computerized cognitive assessment tool in clinical research, but its reliability over short retest intervals has not been thoroughly assessed. This study evaluates the reliability of NIHTB-CB subtests over a 3-week retest interval in participants from an ongoing longitudinal study with biannual NIHTB-CB assessment. Thirteen community-dwelling African American/Black adults aged 65 or older (M = 70.61/SD = 3.36), recruited from the Institute of Gerontology at Wayne State University (R01AG054484; Kavcic, PI) and the Michigan Alzheimer’s Disease Research Center (MADRC, P30AG072931; Paulson, PI), participated. Participants had previously completed NIHTB-CBv2 on iPad tablets every six months for approximately 18 months (M = 17.46/SD = 7.52). The distribution of diagnoses was: 9 normal cognition, 2 non-amnestic MCI and 3 Cognitively Impaired, not MCI. For this study, they now completed the NIHTB-CB twice over a 3-week interval. Reliability was assessed using intraclass correlations (ICCs). Paired t-tests were used to assess overall change in scores over time. All NIHTB-CB ICCs ranged from 0.75 to 0.90, indicating good reliability, except List Sorting Working Memory that had an ICC in the moderate range (0.55). No significant differences were found for any paired t-tests. As clinical trials increasingly use computer-based testing measures, understanding their reliability over short intervals is crucial. Our findings indicate that the NIHTB-CB, when participants are familiar with the test, still shows acceptable retest reliability for both summary indices and subtests over even short retest intervals.

